# Supplementary material for: Functionalisation of Silicone by Drug-Embedded Chitosan Nanoparticles for Potential Applications in Otorhinolaryngology
Source: Materials (Basel). 2019 Mar 13;12(6):847. doi: 10.3390/ma12060847 (PMC6471903; doi:10.3390/ma12060847)
Supplement: Supplementary file 1 [file materials-12-00847-s001.pdf]

materials

MDPI

# Supplementary Materials

## Weibull model and Korsmeyer-Peppas model data

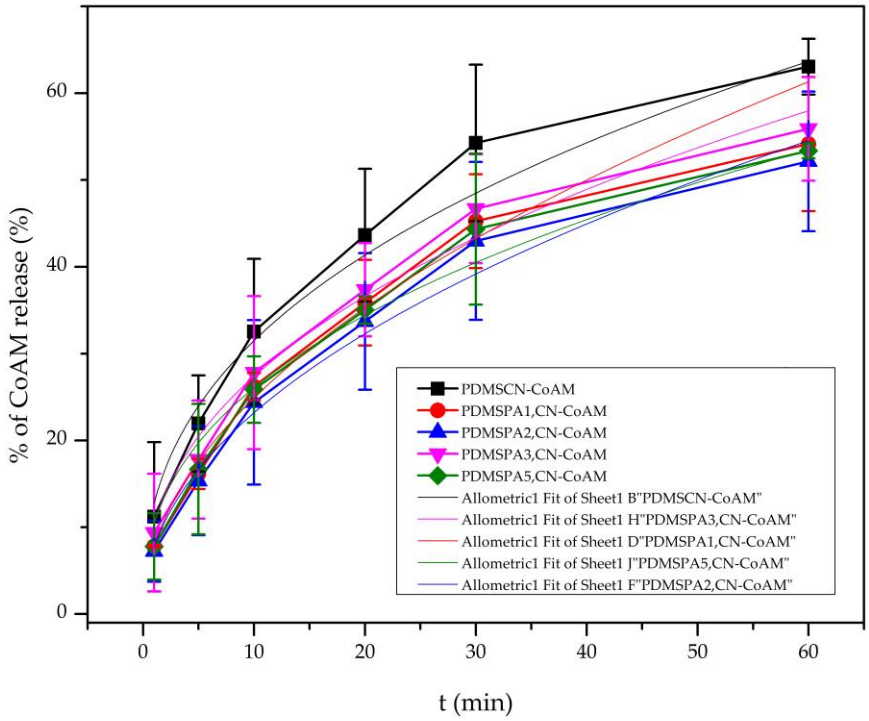


**Figure S1.** Time dependent change in the percentage of the released incorporated drug with Weibull model fitting graph.

**Table S1.** Weibull model fitting data.

| | | | | | |
|---|---|---|---|---|---|
| **Model** | **ExpDec** | | | | |
| **Equation** | **y = A1*exp(-x/t1) + y0** | | | | |
| **Reduced Chi-Sqr** | 1.92182 | 2.29953 | 1.00015 | 1.82172 | 2.72962 |
| **Adj. R-Square** | 0.94070 | 0.97292 | 0.97202 | 0.96527 | 0.98618 |
| | | **Value** | **Standard Error** | | |
| **$PDMS_{CN\text{-}CoAM}$** | y0 | 100.06891 | 4.78741 | | |
| | A1 | -74.48291 | 5.65544 | | |
| | t1 | 115.83608 | 25.00505 | | |
| | k | 0.00863 | 0.00186 | | |
| | tau | 80.29145 | 17.33218 | | |
| **$PDMS_{PA1,\ CN\text{-}CoAM}$** | y0 | 84.51165 | 4.66911 | | |
| | A1 | -78.74108 | 4.63205 | | |
| | t1 | 37.88751 | 5.52422 | | |
| | k | 0.02639 | 0.00385 | | |
| | tau | 26.26162 | 3.82910 | | |
| **$PDMS_{PA2,\ CN\text{-}CoAM}$** | y0 | 98.15534 | 4.03156 | | |
| | A1 | -87.12768 | 4.69160 | | |
| | t1 | 124.53892 | 21.81387 | | |

| | | | |
|---|---|---|---|
| | k | 0.00803 | 0.00141 |
| | tau | 86.32380 | 15.12022 |
| **PDMS$_{PA3, CN-CoAM}$** | y0 | 101.20670 | 2.73233 |
| | A1 | -80.19450 | 4.59340 |
| | t1 | 142.04842 | 18.26431 |
| | k | 0.00704 | $9.05170\times10^{-4}$ |
| | tau | 98.46046 | 12.65985 |
| **PDMS$_{PA5, CN-CoAM}$** | y0 | 99.61290 | 1.62852 |
| | A1 | -80.43160 | 3.61425 |
| | t1 | 109.90672 | 11.42621 |
| | k | 0.00910 | $9.45919\times10^{-4}$ |
| | tau | 76.18153 | 7.92005 |

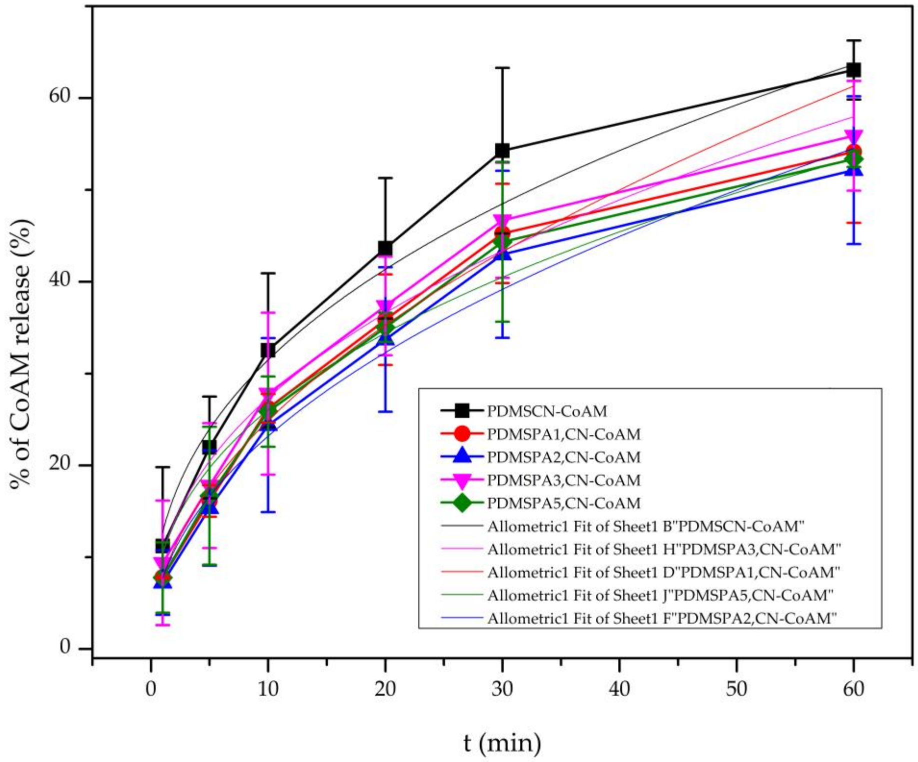


**Figure S2.** Time dependent change in the percentage of the released incorporated drug with Korsmeyer-Peppas model fitting graph.

**Table S2.** Korsmeyer-Peppas model fitting data.

| **Model** | **Allometric** | | | | |
|---|---|---|---|---|---|
| **Equation** | **y =a*x^b** | | | | |
| **Reduced Chi-Sqr** | 0.17949 | 0.60260 | 0.09399 | 0.15151 | 0.24376 |
| **Adj. R-Square** | 0.98650 | 0.98900 | 0.98804 | 0.97963 | 0.99534 |
| | | **Value** | **Standard Error** | | |
| **PDMS$_{CN-CoAM}$** | **a** | 12.74275 | 1.44202 | | |
| | **b** | 0.39287 | 0.02919 | | |
| **PDMS$_{PA1, CN-CoAM}$** | **a** | 7.81056 | 0.11223 | | |
| | **b** | 0.50322 | 0.01350 | | |

| | | | |
|---|---|---|---|
| **PDMS$_{PA2, CN-CoAM}$** | **a** | 7.72702 | 0.78297 |
| | **b** | 0.47723 | 0.02930 |
| **PDMS$_{PA3, CN-CoAM}$** | **a** | 10.42918 | 1.30988 |
| | **b** | 0.41895 | 0.03549 |
| **PDMS$_{PA5, CN-CoAM}$** | **a** | 10.34756 | 0.72216 |
| | **b** | 0.40109 | 0.01759 |
